# Diet quality from mid-life and body composition in older age: findings from a British birth cohort

**DOI:** 10.1017/S0007114524002988

**Published:** 2025-01-28

**Authors:** Leo D. Westbury, Ruth Durdin, Sian M. Robinson, Cyrus Cooper, Rachel Cooper, Kate A. Ward

**Affiliations:** 1 MRC Lifecourse Epidemiology Centre, University of Southampton, Southampton, UK; 2 NIHR Southampton Biomedical Research Centre, University of Southampton and University Hospital Southampton NHS Foundation Trust, Southampton, UK; 3 AGE Research Group, Translational and Clinical Research Institute, Newcastle University, Newcastle upon Tyne, UK; 4 NIHR Newcastle Biomedical Research Centre, Newcastle upon Tyne Hospitals NHS Foundation Trust, Cumbria, Northumberland, Tyne and Wear NHS Foundation Trust and Newcastle University, Newcastle upon Tyne, UK; 5 NIHR Oxford Biomedical Research Centre, University of Oxford, Oxford, UK

**Keywords:** Epidemiology, Diet, Body composition, Lifecourse, Ageing

## Abstract

We investigated associations between ‘healthy dietary pattern’ scores, at ages 36, 43, 53 and 60–64 years, and body composition at age 60–64 years among participants from the MRC National Survey of Health and Development (NSHD). Principal component analyses of dietary data (food diaries) at age 60–64 years were used to calculate diet scores (healthy dietary pattern scores) at each age. Higher scores indicated healthier diets (higher consumption of fruit, vegetables and wholegrain bread). Linear regression was used to investigate associations between diet scores at each age and height-adjusted dual-energy X-ray absorptiometry-measured fat and lean mass measures at age 60–64 years. Analyses, adjusting for sex and other potential confounders (age, smoking history, physical activity and occupational class), were implemented among 692 men and women. At age 43, 53 and 60–64 years, higher diet scores were associated with lower fat mass index (FMI) and android:gynoid fat mass ratio; for example, in fully adjusted analyses, a standard deviation (sd) increase in diet score at age 60–64 years was associated with an SD difference in mean FMI of −0·18 (95 % CI: −0·25, −0·10). In conditional analyses, higher diet scores at ages 43, 53 and 60–64 years (than expected from diet scores at younger ages) were associated with lower FMI and android:gynoid fat mass ratio in fully adjusted analyses. Diet scores at age 36 years had weaker associations with the outcomes considered. No associations regarding appendicular lean mass index were robust after full adjustment. This suggests that improvements in diet through adulthood are linked to beneficial effects on adiposity in older age.

Body composition, including lean and fat mass, is an important determinant of musculoskeletal health in older age. The loss of muscle mass, strength and function characterises sarcopenia, which is associated with disability, morbidity and poorer bone health including increased fracture risk^(1)^. Greater fat mass is associated with poorer musculoskeletal health, including reduced muscle quality and greater loss of lean mass^(2)^. Adipose tissue is a source of inflammatory cytokines and adipokines, and higher levels of inflammation have been associated with greater loss of muscle strength^(3)^. Given the high burden of poorer musculoskeletal health in older age for both individuals and healthcare systems, it is a priority to further understand the determinants of body composition in older age.

In older adults, relationships have been demonstrated between individual nutrients and muscle mass and function; however, given the complexities of diet, there is increasing recognition of the importance of understanding dietary patterns as a whole^(4)^. To date, most studies have investigated cross-sectional relationships between diet and body composition in older adults^(5–7)^. However, it is also important to further understand how diet across adulthood is associated with body composition in older age, and the potential for earlier interventions. The association between change in diet quality and change in weight over a 20-year period was investigated in a study comprising 50 603 women in the Nurses’ Health Study, 22 973 men in the Health Professionals Follow-Up Study and 72 495 younger women from the Nurses’ Health Study II^(8)^. This study found that improvement in diet quality was associated with lower rates of long-term weight gain. Stronger associations were observed among younger individuals, and those who were overweight and obese. However, overall measures of body weight are unable to distinguish fat and lean mass, which individually make distinct contributions to musculoskeletal health. Indeed, to date, few studies have investigated longitudinal relationships between diet quality and these different components of body composition^(9–11)^.

These previous studies that have investigated relationships between diet quality and body composition have used a variety of diet scores, including *a priori* scoring methods (based on the healthiness of nutrients or food items) and data-driven approaches; many of these studies utilised more than one diet score. Different types of diet scores included in these studies were those that reflect: extent of alignment with a Mediterranean diet^(5,8,9)^; diet quality according to compliance with dietary guidelines^(6,7,9–11)^, such as the Australian Recommended Food Score (ARFS)^(10)^; extent to which an individual’s diet lowers the risk of chronic disease (Dietary Approaches to Stop Hypertension (DASH) score^(8,9)^) or level of inflammation (Dietary Inflammatory Index^(10,11)^); and alignment with some dietary pattern ascertained using principal component analysis (PCA)^(10,11)^. The current study used a diet score ascertained using PCA for several reasons. This diet score had been used previously in the same cohort and was found to be strongly correlated with key food items^(12)^ and nutrients (online Supplementary Table 1) which are known to be markers of diet quality. Furthermore, PCA-derived diet scores are based on actual dietary patterns observed in the cohort which explain the most variation in diet, as opposed to the subjective criteria of *a priori* scoring systems that may differ between countries and settings.

Using data from a British birth cohort study, the aim of this study was to investigate associations between diet quality between the ages of 36 and 60–64 years and body composition parameters at age 60–64 years.

## Methods

### Study sample

The MRC National Survey of Health and Development (NSHD) is a longitudinal study based on a sample of 5362 births occurring in March 1946 across England, Scotland and Wales^(13)^. Participants in this study have been extensively phenotyped on multiple occasions from 1946 to 2006–2010 via home visits by research nurses, postal questionnaires and clinic visits. The information ascertained at each follow-up from 1946 until 2006–2010 has been described in detail previously^(14)^. By the 2006–2010 follow-up, 594 participants had withdrawn from the study, 567 had emigrated, 718 had died and 320 were lost to follow-up. Of those remaining, 2229 (78 % of those invited) were assessed at the 2006–2010 follow-up. At this follow-up, participants were invited to attend one of six clinical research facilities across the UK for clinical assessments; the option of a home visit by a research nurse was offered if the study member refused or was unable to attend the clinical research facility^(15)^. Overall, 1690 (76 %) were assessed at clinic and 539 (24 %) were assessed at home^(13,15)^.

### Dietary assessment

Diet was assessed using prospective 5-day food diaries completed by participants at ages 36, 43, 53 and 60–64 years^(16,17)^. Food and drink items consumed were recorded using household measures. Diaries included notes and images to aid estimation of portion size. Each item was allocated to one of forty-five food groups based on similarity of food type and nutritional composition^(12)^. Mean consumption of each food group (g/day) across the 5 days was calculated for participants at each age. The same forty-five food groups were used at each age.

Using Stata, release 17 (StataCorp), a PCA of the consumption (g/day) of the forty-five food groups at age 60–64 years was performed using the correlation matrix of these variables as they had markedly different ranges. The first component was characterised by higher consumption of fruit, vegetables and wholegrain bread, and lower consumption of white bread, potato products, added sugar and processed meat. Other components were not considered as their interpretation was unclear in terms of the dietary pattern that they reflected. Diet scores, reflecting compliance with this ‘healthier’ dietary pattern, were calculated at ages 36, 43, 53 and 60–64 years by multiplying the consumption of each of the forty-five food groups by its PCA coefficient and summing these over all food groups. The PCA coefficients derived at age 60–64 years were used to calculate diet scores at each age in adulthood when diet quality was assessed (36, 43, 53 and 60–64 years) to ensure that diet quality was measured on the same scale at each age; higher scores indicated healthier diets. Diet scores were interpreted as a measure of diet ‘quality’^(12,18)^. Although broadly similar coefficients for the food groups were obtained when the PCA was performed at the different ages when diet quality was assessed, as shown previously^(12)^, the dietary pattern reflected by the first component was most aligned with a healthy dietary pattern for the PCA performed at age 60–64 years. For this reason, the PCA coefficients at this time point were used to derive diet scores at each age. Overall, 988 (53 %) participants completed food diaries (for at least 3 days) at all the following ages: 36, 43, 53 and 60–64 years. The use of PCA to derive diet quality scores in this cohort has been described in more detail previously^(12)^.

The diet quality score used in the current study was positively associated with key nutrients (excluding supplements) which are important for maintaining a healthy diet (online Supplementary Table 1), and marked differences in the consumption of food items known to reflect diet quality have been shown according to quarters of this diet score^(12)^.

### Ascertainment of body composition measures at age 60–64 years

Body composition measures were obtained for 1658 (98 %) clinic participants using a QDR 4500 Discovery dual-energy X-ray absorptiometry (DXA) scanner (Hologic Inc.); quality assurance procedures have been described previously^(19)^. Whole body, android and gynoid fat mass as well as appendicular lean mass (ALM) measures were obtained. Body composition data for the head has been known to affect measures relating to soft tissue, so these data were excluded in the assessment of these whole body measures^(20,21)^.

Fat mass index (FMI) (whole body fat mass (kg)/height (m^1·2^) was derived as in previous analyses^(20,22)^. ALM index was derived by dividing ALM (kg) by the square of height (m). Android (abdominal):gynoid (hip) fat mass ratio was also derived.

### Ascertainment of other participant characteristics

At the age of 53 years, participants’ own occupational class, as an indicator of socio-economic position, was categorised using the Registrar General’s Social classification: I (Professional); II (Intermediate); IIINM (Skilled Non-manual); IIIM (Skilled Manual); IV (Partly Skilled); and V (Unskilled).

The participant characteristics in this paragraph were all ascertained at age 60–64 years. Height and weight were measured using standard protocols by trained nurses. Self-reported smoking status (never/ex/current) was categorised into ever smokers and never smokers. Leisure time physical activity was assessed by questionnaire; participants were asked how often in the previous month they had participated in any sports, vigorous leisure activities or exercises; participants were categorised as follows: inactive (no participation); moderately active (participated in relevant activities 1–4 times per month); and active (participated in relevant activities five or more times per month).

### Statistical analysis

Participant characteristics were described using summary statistics. An overall Adult Diet Quality (ADQ) score was calculated for each participant using the following process: the diet quality score at age 60–64 years was categorised into quarters and the boundary values of these quarters were recorded; for each participant, these boundary values were then used to attribute a score to their continuous diet quality score at ages 36, 43, 53 and 60–64 years (1 = lowest quarter, 2 = second, 3 = third and 4 = highest quarter). These points were summed across the four ages; scores ranged from 4 (a participant with poor diet quality across all ages in adulthood) to 16 (a participant with high diet quality across all ages in adulthood).

Diet quality scores at each age and body composition measures were standardised (sex-specific) in models so that they had a mean of 0 and a sd of 1 within each sex to ensure comparability of effect sizes. Linear regression was used to examine the associations between diet quality scores at different ages and ADQ in relation to FMI, ALM index and android:gynoid fat mass ratio. Sex-adjusted and fully adjusted models, accounting for sex, age at follow-up, smoking history, physical activity and occupational class, were implemented as these are established correlates of both diet quality and body composition and, therefore, are likely to confound the associations of interests. Fully adjusted models for android:gynoid fat mass ratio also accounted for height. All models for ALM index were adjusted for FMI as these indices are positively correlated with each other, so this adjustment ensures that any associations identified between diet quality and ALM index were independent of FMI.

Conditional models were used to examine whether higher diet quality than expected at ages 43, 53 and 60–64 years, given diet quality at earlier ages, was associated with differences in body composition measures. To construct these models, residuals (representing conditional diet quality scores), were obtained from models with diet quality scores at each age as the outcome and diet quality scores at all previous ages as the set of exposures^(23)^. Each body composition parameter was then regressed on all conditional diet quality scores with adjustment for diet quality at age 36 years and the sets of adjustments outlined above. This approach was implemented as the diet quality scores at each time point were positively correlated with each other as described previously^(12)^, illustrating that diet quality tracked over time in this cohort.

Of the 988 participants with diet scores at every adult assessment, 721/988 (73 %) had complete data for the body composition outcomes and 692/988 (70 %) had complete data for the body composition outcomes and covariates. Therefore, the analysis sample was restricted to the group of 692 participants with complete data. This approach was taken as some of the statistical methods implemented require participants to have diet scores available at every adult assessment, and complete data for the outcomes and covariates ensure that differences in associations are not due to different individuals being included in different statistical models. Assuming a significance level of *P*< 0·05, 80 % power and a standardised predictor and outcome used in a simple linear regression model, this sample size results in a minimum detectable effect size of 0·11 (0·11 sd difference in outcome per 1 sd increase in predictor). To examine whether associations in the analysis sample were generalisable to the wider cohort, sensitivity analyses were conducted among the group of 1553 participants with diet scores at one or more time points and data on at least one body composition outcome.

Formal tests for sex interaction illustrated that relationships between diet quality scores and body composition outcomes were similar among men and women, so men and women were pooled and analyses were adjusted for sex. All analyses were conducted using Stata, release 17 (StataCorp).

## Results

### Participant characteristics

The characteristics of the analysis sample are presented in Table 1. Mean age at clinic visit was 63 years among men and women. Mean diet quality was higher among women than men at each age and increased from age 36 to age 60–64 years among both men and women. Mean FMI was greater among women, whereas mean ALM index and android:gynoid fat mass ratio were greater among men.

**Table 1.** Participant characteristics of the analysis sample (Mean values and standard deviations; numbers and percentages)

Participant characteristic	Men (*n* 310)		Women (*n* 382)	
	Mean	sd	Mean	sd
Age at clinic visit (years)	63·1	1·1	63·2	1·1
Height (m)	1·75	0·07	1·62	0·06
Weight (kg)	84·6	13·1	70·8	12·6
BMI (kg/m^2^)	27·5	4·0	26·8	4·7
	*n*	%	*n*	%
BMI categories (kg/m^2^)*				
Underweight (BMI < 18·5)	1	0·3 %	4	1·0 %
Normal (18·5 ≤ BMI < 25)	89	28·7 %	152	39·8 %
Overweight (25 ≤ BMI < 30)	137	44·2 %	138	36·1 %
Obese (BMI ≥ 30)	83	26·8 %	88	23·0 %
Ever smoked regularly*	210	67·7 %	222	58·1 %
Leisure time physical activity participation*				
None	186	60·0 %	202	52·9 %
1–4 times/month	46	14·8 %	76	19·9 %
5 or more times/month	78	25·2 %	104	27·2 %
	Mean	sd	Mean	sd
Diet scores at various ages (years)†				
36	–2·0	1·2	–1·5	1·2
43	–1·6	1·7	–0·8	1·5
53	–0·8	1·7	0·1	1·6
60–64	0·0	1·9	0·7	1·6
	*n*	%	*n*	%
Occupational class*				
I – Professional	44	14·2 %	9	2·4 %
II – Intermediate	144	46·5 %	170	44·5 %
III – Skilled (non-manual)	33	10·6 %	141	36·9 %
III – Skilled (manual)	62	20·0 %	23	6·0 %
IV – Partly skilled	23	7·4 %	29	7·6 %
V – Unskilled	4	1·3 %	10	2·6 %
	Mean	sd	Mean	sd
Fat mass index (kg/m^1·2^)	12·0	3·8	15·6	4·7
Appendicular lean mass index (kg/m^2^)	8·0	0·9	6·1	0·8
Android:gynoid fat mass ratio	0·64	0·15	0·44	0·12

* *n* (%).

† Diet quality scores defined using food consumption data collected at each age and coefficients from a principal component analysis of the dietary data collected at 60–64 years; higher scores indicate healthier diets.

Men and women included in the analytical sample had lower BMI (*P*< 0·03) and were more likely to have engaged in physical activity at least once per month (*P*< 0·03) in comparison with those who were assessed at age 60–64 years but were not included due to having incomplete data regarding the variables of interest; only women in the analytical sample were more likely to have never smoked (*P*= 0·003). Compared with those not included, men and women in the analysis sample had lower mean android:gynoid fat mass ratio (*P*< 0·03); FMI was lower among women only (*P*< 0·001); and there was no difference in ALM index among either sex (*P*> 0·1).

### Relationship between diet quality in adulthood and measures of body composition

In sex- and fully adjusted analyses, higher ADQ scores and higher diet quality scores at ages 43, 53 and 60–64 years were each associated with lower FMI and android:gynoid fat mass ratio (Table 2). For example, a 1 sd increase in diet score at age 60–64 years was associated with a difference in mean FMI of −0·18 sd (95 % CI −0·25, −0·10) in fully adjusted analyses. Higher ADQ scores and higher diet scores at ages 43 and 60–64 years were associated with higher ALM index after adjustment for sex and FMI, but these associations were not robust to further adjustment.

**Table 2.** sd difference in body composition outcomes at 60–64 years per sd increase in diet score* at each age and per unit increase in adult score

Age (years)	M‡	Fat mass index			ALM index			Android:gynoid fat mass ratio		
		Estimate	95 % CI	*P*	Estimate	95 % CI	*P*	Estimate	95 % CI	*P*
36	1	–0·03	–0·10, 0·04	0·438	0·03	–0·03, 0·09	0·337	–0·08	–0·16, −0·01	0·028
	2	–0·01	–0·09, 0·06	0·772	0·02	–0·04, 0·08	0·607	–0·07	–0·14, 0·01	0·080
43	1	–0·12	–0·19, −0·04	0·002	0·07	0·01, 0·13	0·021	–0·17	–0·25, −0·10	<0·001
	2	–0·10	–0·18, −0·02	0·011	0·06	0·00, 0·12	0·063	–0·16	–0·24, −0·08	<0·001
53	1	–0·13	–0·20, −0·06	<0·001	0·04	–0·02, 0·10	0·185	–0·22	–0·29, −0·15	<0·001
	2	–0·11	–0·19, −0·04	0·003	0·02	–0·03, 0·08	0·418	–0·21	–0·28, −0·14	<0·001
60–64	1	–0·19	–0·27, −0·12	<0·001	0·07	0·01, 0·13	0·023	–0·21	–0·28, −0·13	<0·001
	2	–0·18	–0·25, −0·10	<0·001	0·05	–0·01, 0·11	0·109	–0·20	–0·28, −0·13	<0·001
ADQ†	1	–0·05	–0·07, −0·02	<0·001	0·02	0·00, 0·04	0·042	–0·07	–0·09, −0·05	<0·001
	2	–0·04	–0·07, −0·02	0·001	0·01	–0·01, 0·04	0·146	–0·07	–0·09, −0·04	<0·001

ALM, appendicular lean mass.

* Diet quality scores defined using food consumption data collected at each age and coefficients from a principal component analysis of the dietary data collected at 60–64 years of age; higher scores indicate healthier diets.

† Adult Diet Quality (ADQ) scores, where individuals’ scores were coded from 1 (lowest quartile) to 4 (highest quartile) between age 36 to age 60–64 years and summed to yield a score from 4 to 16 (quartile boundaries based on diet score at 60–64 years of age).

‡ Model 1: adjusted for sex; Model 2: adjusted for sex, age at follow-up, smoking history, physical activity and occupational class (android:gynoid fat mass ratio was also adjusted for height). All models for ALM index were also adjusted for fat mass index.

In sensitivity analyses, among the wider group of participants with diet scores at one or more time points and data on at least one body composition outcome, relationships were similar for FMI and android:gynoid fat mass ratio as outcomes; fully adjusted associations between higher diet scores at age 43 and 60–64 years in relation to greater ALM index were also robust (online Supplementary Table 2).

Because there was tracking of diet quality across adulthood, conditional models were used to explore whether higher diet quality than predicted, based on earlier diet quality scores, was associated with body composition outcomes (Table 3). Using these models, higher diet quality than expected at age 43, 53 and 60–64 years, when taking into account earlier diet quality, was associated with lower FMI and android:gynoid fat mass ratio in both sex- and fully adjusted analyses.

**Table 3.** sd difference in body composition outcomes at 60–64 years per sd increase in conditional diet quality at each age

Age (years)	M*	Fat mass index			ALM index			Android:gynoid fat mass ratio		
		Estimate	95 % CI	*P*	Estimate	95 % CI	*P*	Estimate	95 % CI	*P*
43	1	–0·12	–0·19, −0·05	0·001	0·06	0·01, 0·12	0·032	–0·15	–0·22, −0·08	<0·001
	2	–0·11	–0·18, −0·04	0·004	0·06	0·00, 0·11	0·060	–0·14	–0·21, −0·07	<0·001
53	1	–0·09	–0·16, −0·01	0·020	0·00	–0·06, 0·06	0·962	–0·15	–0·22, −0·08	<0·001
	2	–0·08	–0·15, −0·01	0·029	–0·01	–0·06, 0·05	0·801	–0·15	–0·22, −0·08	<0·001
60–64	1	–0·14	–0·21, −0·07	<0·001	0·04	–0·02, 0·10	0·149	–0·08	–0·15, 0·00	0·038
	2	–0·13	–0·20, −0·05	0·001	0·03	–0·03, 0·09	0·290	–0·08	–0·15, 0·00	0·036

ALM, appendicular lean mass.

Estimates represent the sd difference in body composition outcomes per sd increase in diet quality at each age, conditional on diet quality at all previous ages.

* Model 1: adjusted for sex; Model 2: adjusted for sex, age at follow-up, smoking history, physical activity and occupational class (android:gynoid fat mass ratio was also adjusted for height). All models for ALM index were also adjusted for fat mass index.

## Discussion

Using dietary data ascertained prospectively over 30 years of follow-up in the MRC NSHD, we have investigated relationships between diet quality (between age 36 and 60–64 years) and body composition at age 60–64 years. Higher quality diets were characterised by higher consumption of fruit and vegetables and wholegrain bread, and lower consumption of white bread, potato products, added sugar and processed meat and were associated with lower FMI and android:gynoid fat mass ratio at age 60–64 years. These relationships were found when considering diet at ages 43, 53 and 60–64 years individually as well as for an index of overall diet quality in adulthood and remained after adjustment for potential confounders. Associations between higher diet quality at ages 43 and 60–64 years and higher ALM index were observed in initial models but were not robust to adjustment. In sensitivity analyses, comprising a larger group of participants with diet scores at one or more time points, robust associations between diet quality at ages 43 and 60–64 years in relation to ALM index were observed. We also found that having a higher quality diet than expected at age 43, 53 or 60–64 years, based on diet quality earlier in adulthood, was associated with lower FMI and android:gynoid fat mass ratio, suggesting that improvements to diet may be beneficial and are linked to differences in body composition in early older age.

Although sex-adjusted associations were observed for diet quality at age 36 years in relation to android:gynoid fat mass ratio in the main analysis, and in relation to both ALM index and android:gynoid fat mass ratio in the supplementary analysis, these associations were weaker compared with those using diet quality scores from later time points. A potential explanation for this is that age 36 years is when the duration between the diet quality exposure and the body composition outcomes was greatest. This is supported by the fact that the strength of associations and effect sizes were generally greater for the diet quality scores measured closer in time to the ascertainment of the body composition outcomes.

Relatively few studies have investigated longitudinal relationships between diet and body composition. However, some studies have implemented a similar approach to our analyses in that they have examined longitudinal changes in diet quality in relation to measures of adiposity or body composition. For example, in a study involving participants of the Nurses’ Health Study, the Health Professionals Follow-Up Study and the Nurses’ Health Study II, diet quality was assessed via three indices (the Alternative Mediterranean Diet (aMED), the Alternative Healthy Eating Index-2010 (AHEI-2010) and the DASH); based on data collected every 4 years, overall improvements in diet quality were associated with less weight gain over a 20-year period^(8)^. These results align with associations reported in our study between conditional change in diet quality (higher diet quality than expected from earlier diet quality) and lower adiposity; for consistency, we did not examine longitudinal change in weight as an outcome as the body composition outcomes considered were only available at a single time point.

In addition, various studies have reported longitudinal associations between diet quality and fat mass distribution. For example, in the Multiethnic Cohort Study, higher diet quality at baseline as assessed by four indices (the Healthy Eating Index-2010 (HEI-2010), AHEI-2010, aMED and DASH) was associated with lower adiposity measures including MRI-based visceral adiposity tissue area and DXA-based total body fat at 20-year follow-up ^(9)^. These results are therefore similar to those which we present showing an inverse association between diet quality and android:gynoid fat mass ratio, which is a marker of central adiposity. Interestingly, this study also reported a lack of association between diet quality and DXA-based muscle mass index, which is comparable to the results presented for ALM index in our study.

In contrast to our results, longitudinal associations between higher diet quality and reduced loss of lean mass have been reported in other studies. In men in the Geelong Osteoporosis Study, indices of diet quality included the ARFS, the Dietary Inflammatory Index and three PCA-derived indices (plant-focused, Western and traditional diets). Lower Dietary Inflammatory Index score (indicating diets likely to encourage lower levels of chronic inflammation) and higher traditional diet score (characterised by higher consumption of red, white and processed meats, unprocessed fish, fruits and vegetables, wholegrain cereals, nuts and discretionary foods (cakes and biscuits)) were associated with a smaller decrease in ALM index at the 15-year follow-up^(10)^. In women in the Geelong Osteoporosis Study, a higher ARFS was associated with greater ALM index after 5 years ^(11)^. The traditional dietary pattern and ARFS described by this study share similarities to the diet quality score used in our study; however, there are some differences in terms of the foods most important for determining these scores.

We did not find associations between diet quality and ALM index after adjustment in the main analysis. Interestingly, higher diet quality was associated with better physical performance as previously described in the MRC NSHD ^(12)^. These results may be explained by loss of muscle strength with age occurring before loss of muscle mass ^(24)^, the former potentially being more important regarding physical performance. This explanation could be particularly relevant to the MRC NSHD given that all participants were born in 1946 and, therefore, were of a similar age at the 2006–2010 follow-up. Among women in the Helsinki Birth Cohort Study, there were associations between a higher Nordic Diet Score (NDS) (based on nine components: fruits, vegetables, cereals, low-fat and fat-free milk, fish, polyunsaturated: saturated fatty acid ratio, red/processed meat intake, total fat, alcohol intake) and greater muscle strength at 10-year follow-up ^(25)^. In agreement with our study, the NDS was also not associated with muscle mass (measured via bioelectrical impedance analysis) in men or women. These results highlight the need for other aspects of muscle quality, such as intra- and inter-muscular fat, to be examined in relation to diet quality in earlier life as muscle quality may be a mechanism by which higher diet quality is related to improved physical performance in older age.

A strength of our study is the use of dietary data prospectively ascertained at four time points across adulthood, meaning that it was possible to assess both diet across adulthood and change in diet quality in relation to body composition. In addition, diet scores at age 36, 43 and 53 years were based on PCA coefficients generated at age 60–64 years, which ensured diet quality was measured on the same scale at each age. We also used measures of body composition obtained using DXA, which provide greater accuracy of different body compartments than anthropometric measurements. However, a limitation is that the analysis sample comprised only individuals with diet scores at every adult assessment, and with complete data for the body composition outcomes and adjustments; this resulted in a considerable decrease in the size of the analysis sample. Furthermore, there were differences in the descriptive characteristics of the analysis sample when compared with the wider group of MRC NSHD participants who were not included which may limit the generalisability of findings. However, analyses were internal to the analysis sample and major bias would only be introduced if associations of interest differed systematically between the analysis sample and the group of participants who were not included. Furthermore, we found similar relationships in sensitivity analyses among the maximal sample of participants with diet scores at one or more time points in adulthood. Although there are limitations to the use of self-reported dietary data which have been previously described, these may be relatively less important for overall dietary patterns. A number of studies have shown that comparable patterns are defined when using different dietary assessment methods and the validity of dietary patterns identified using food diaries has been shown ^(12)^. Another limitation is that deriving the overall adult diet score by assigning scores to quarters of the continuous diet quality score results in a loss of information and does not distinguish between participants with an average diet quality across all time points and those with a diet quality that fluctuates between high and low levels over time. Finally, it is important to acknowledge that residual confounding, whereby the observed associations are due to measured and unmeasured confounders which were not taken into account, is a potential explanation of the findings reported. Similarly, most adjustments used in the analysis were ascertained at age 60–64 years as this was the time point when the body composition outcomes were ascertained; there is a possibility that changes in these characteristics over time could have influenced the associations observed, for example, there is evidence that changes in lifestyle factors occurred in this cohort from ages 53 to 60–64 years ^(26)^.

In conclusion, we have demonstrated associations between higher diet quality – both at individual ages and across adulthood overall – and reduced fat mass in older age. Modest associations between higher diet quality and greater lean mass were also observed, but these were less robust. The potential implications of these findings are that better diet quality, even in earlier adulthood, may lead to a reduced risk of adiposity and sarcopenia in older age, and therefore healthier ageing in general.

## Supporting information

Westbury et al. supplementary material 1 Westbury et al. supplementary material

Westbury et al. supplementary material 2 Westbury et al. supplementary material

## Supplementary material

For supplementary material/s referred to in this article, please visit https://doi.org/10.1017/S0007114524002988
