# Supplementary material for: Diet quality from mid-life and body composition in older age: findings from a British birth cohort
Source: Br J Nutr. 2024 Dec 4;133(2):262–8. doi: 10.1017/S0007114524002988 (PMC11813619; doi:10.1017/S0007114524002988)
Supplement: Westbury et al. supplementary material 1 — Westbury et al. supplementary material [file S0007114524002988sup001.docx]

| **Supplementary Table 1: Cross-sectional associations between the diet quality score and nutrient intakes (excluding supplements) among men and women at age 60-64** | | | | |  |
| --- | --- | --- | --- | --- | --- |
|  |  |  |  |  |  |
|  |  |  |  |  |  |
| **Nutrient** | **Men** | | **Women** | |  |
|  | **Spearman correlation coefficient** | **P-value** | **Spearman correlation coefficient** | **P-value** |  |
| Protein | 0.27 | <0.001 | 0.22 | <0.001 |  |
| Fat | 0.09 | 0.027 | 0.00 | 0.984 |  |
| Saturated Fatty Acids | 0.02 | 0.689 | -0.13 | <0.001 |  |
| Carbohydrate | 0.28 | <0.001 | 0.25 | <0.001 |  |
| Fibre | 0.70 | <0.001 | 0.66 | <0.001 |  |
| Retinol | 0.03 | 0.374 | -0.04 | 0.325 |  |
| Carotene | 0.49 | <0.001 | 0.46 | <0.001 |  |
| Thiamin | 0.36 | <0.001 | 0.29 | <0.001 |  |
| Riboflavin | 0.30 | <0.001 | 0.26 | <0.001 |  |
| Vitamin B6 | 0.30 | <0.001 | 0.32 | <0.001 |  |
| Folate | 0.41 | <0.001 | 0.45 | <0.001 |  |
| Vitamin B12 | 0.23 | <0.001 | 0.22 | <0.001 |  |
| Vitamin C | 0.66 | <0.001 | 0.61 | <0.001 |  |
| Vitamin D | 0.23 | <0.001 | 0.25 | <0.001 |  |
| Vit E | 0.42 | <0.001 | 0.43 | <0.001 |  |
| Calcium | 0.33 | <0.001 | 0.34 | <0.001 |  |
| Iron | 0.43 | <0.001 | 0.38 | <0.001 |  |
| Zinc | 0.31 | <0.001 | 0.24 | <0.001 |  |
|  |  |  |  |  |  |
| Correlations examined within the maximum available sample (1553 participants with data on diet scores at one or more time-points and data on at least one body composition outcome) | | | | |  |
|  |  |  |  |  |  |

| **Supplementary Table 2: SD difference in body composition outcomes at 60-64 years per SD increase in diet score^1^ at each age among the maximum available sample (1553 participants with data on diet scores at one or more time-points and data on at least one body composition outcome)** | | | | | | | |
| --- | --- | --- | --- | --- | --- | --- | --- |
|  |  |  | |  | |  | |
| **Age (years)** | **M^2^** | **Fat mass index** | | **ALM index** | | **Android: gynoid**  **fat mass ratio** | |
|  |  | **Estimate (95%CI)** | **P** | **Estimate (95%CI)** | **P** | **Estimate (95%CI)** | **P** |
| *36* | ***1*** | -0.03 (-0.09,0.03) | 0.294 | 0.05 (0.00,0.09) | 0.048 | -0.09 (-0.15,-0.03) | 0.003 |
|  | ***2*** | 0.01 (-0.05,0.07) | 0.748 | 0.03 (-0.03,0.08) | 0.330 | -0.06 (-0.12,0.00) | 0.056 |
| *43* | ***1*** | -0.11 (-0.17,-0.05) | <0.001 | 0.06 (0.01,0.11) | 0.016 | -0.15 (-0.21,-0.09) | <0.001 |
|  | ***2*** | -0.09 (-0.15,-0.03) | 0.003 | 0.05 (0.00,0.10) | 0.046 | -0.14 (-0.20,-0.08) | <0.001 |
| *53* | ***1*** | -0.15 (-0.21,-0.09) | <0.001 | 0.05 (0.00,0.10) | 0.055 | -0.22 (-0.27,-0.16) | <0.001 |
|  | ***2*** | -0.14 (-0.20,-0.07) | <0.001 | 0.03 (-0.02,0.08) | 0.257 | -0.20 (-0.26,-0.14) | <0.001 |
| *60-64* | ***1*** | -0.23 (-0.28,-0.17) | <0.001 | 0.07 (0.03,0.12) | 0.001 | -0.21 (-0.27,-0.16) | <0.001 |
|  | ***2*** | -0.21 (-0.27,-0.16) | <0.001 | 0.06 (0.01,0.11) | 0.012 | -0.19 (-0.25,-0.14) | <0.001 |
| SD: standard deviation; P: P-value; ALM: appendicular lean mass  ^1^Diet quality scores defined using food consumption data collected at each age and coefficients from a principal component analysis of the dietary data collected at 60-64 years; higher scores indicate healthier diets. ^2^Model 1: adjusted for sex; Model 2: adjusted for sex, age at follow-up, smoking history, physical activity and occupational class (android: gynoid fat mass ratio was also adjusted for height). All models for ALM index were also adjusted for fat mass index. | | | | | | | |
